# Challenges and opportunities for checkpoint blockade in T-cell lymphoproliferative disorders

**DOI:** 10.1186/s40425-016-0201-6

**Published:** 2016-12-20

**Authors:** Tycel Phillips, Sumana Devata, Ryan A. Wilcox

**Affiliations:** 1 Department of Internal Medicine, Division of Hematology and Oncology, University of Michigan, Ann Arbor, MI USA; 2 University of Michigan Comprehensive Cancer Center, 4310 Cancer Center, 1500 East Medical Center Drive, Ann Arbor, MI 48109 USA

**Keywords:** Checkpoint blockade, Peripheral T-cell lymphoma, Cutaneous T-cell lymphoma, PD-L1

## Abstract

The T-cell lymphoproliferative disorders are a heterogeneous group of non-Hodgkin’s lymphomas (NHL) for which current therapeutic strategies are inadequate, as most patients afflicted with these NHL will succumb to disease progression within 2 years of diagnosis. Appreciation of the genetic and immunologic landscape of these aggressive NHL, including PD-L1 (B7-H1, CD274) expression by malignant T cells and within the tumor microenvironment, provides a strong rationale for therapeutic targeting this immune checkpoint. While further studies are needed, the available data suggests that responses with PD-1 checkpoint blockade alone will unlikely approach those achieved in other lymphoproliferative disorders. Herein, we review the unique challenges posed by the T-cell lymphoproliferative disorders and discuss potential strategies to optimize checkpoint blockade in these T-cell derived malignancies.

## Background

The resurgent interest in the tumor microenvironment, particularly its role in immunosurveillance, and cancer immunotherapy in the past several years may be largely attributed to the successes achieved with immune-checkpoint blockade (CPB) in multiple solid tumors [1]. Like many solid tumors, most non-Hodgkin’s lymphomas (NHL) are, to varying degrees [2], infiltrated by lymphoid- and myeloid-derived cell subsets that contribute to either immune evasion or immunosurveillance [3]. Genomic alterations involving the PD-L1 (B7-H1, CD274) locus, particularly gene amplification, lead to significant PD-L1 expression in subsets of aggressive B-cell NHL [4–6], while lymphoma-associated macrophages within the tumor microenvironment are an abundant source of PD-L1 in others [7, 8]. Evidence implicating PD-L1 in immune evasion among common B-cell NHL, in conjunction with the high response rates, as high as 87%, observed in relapsed or refractory Hodgkin’s lymphoma with PD-1 CPB [9–11], provides a strong rationale for this approach in B-cell NHL. While many studies are ongoing, response rates exceeding ≈ 33% have been observed in both diffuse large B-cell and follicular lymphomas [12–14].

In contrast to these more common B-cell NHL which are curable or controllable with the existing smorgasbord of therapeutic options, including immunochemotherapy and targeted or immunomodulatory agents, T-cell derived NHL, which account for ≈ 10% of NHL in North America, remain an unmet need, as most patients afflicted with these aggressive mature (peripheral) T-cell lymphomas will succumb to their disease within 2 years of diagnosis [15, 16]. Among peripheral T-cell lymphoma (PTCL) patients ineligible for high-dose therapy and autologous stem-cell transplantation at the time of relapse or progression, median overall survival is less than 6 months [17, 18]. The long-term outlook for patients with advanced-stage cutaneous T-cell lymphomas (CTCL) is similarly discouraging, as durable remissions are rare with existing therapies [19], and median overall survival for those with nodal and/or visceral organ involvement is 1–2 years [20]. Novel therapeutic strategies are urgently needed and clinical trial participation is encouraged for patients afflicted with these T-cell derived NHL.

In a large (*n* = 155) CTCL/PTCL series [21], PD-L1 was expressed by lymphoma cells in 27% of CTCL and 15% of PTCL, but PD-L1 expression within the tumor microenvironment was more common, being observed in 73% and 39% of CTCL and PTCL cases, respectively. Recently described subsets of PTCL, not otherwise specified (PTCL, NOS), the most common PTCL subtype in North America [16], produce an abundance of interferon-γ, a potent inducer of PD-L1 expression [22, 23]. Approximately 25% of adult T-cell leukemia/lymphomas (ATLL), a rare PTCL subtype in most of North America, highly express PD-L1 due to the aberrant truncation of the 3′ untranslated region of PD-L1 mRNA, leading to increased stability of the PD-L1 transcript [24]. Alternatively, translocations culminating in the expression of an NPM-ALK fusion protein in ALK^+^ anaplastic large cell lymphomas (ALCL) lead to STAT3-dependent PD-L1 expression [reviewed in [25]]. As responses to PD-1/PD-L1 CPB are associated with PD-L1 expression in other tumors, these observations reasonably contributed to optimism for CPB in these T-cell derived NHL. The responses observed to date with this strategy, while encouraging, certainly do not approach those achieved in Hodgkin’s lymphoma, and may suggest that CPB in these NHL will require further optimization in future studies. Herein, we will review the limited clinical data available to date, discuss the unique challenges posed by the T-cell derived NHL, and suggest strategies for optimization of CPB in these less common NHL.

### Experience with CPB in CTCL/PTCL

While durable remissions with conventional chemotherapy are rarely achieved in relapsed/refractory T-cell NHL [17–19], durable remissions are achieved with immunomodulatory therapies, including extracorporeal photopheresis (ECP) and interferon-α [reviewed in [26]]. While largely anecdotal, these observations suggest that host immunity, when properly harnessed, can lead to durable responses in selected patients. These observations, coupled with high-level PD-L1 expression in a substantial minority of patients, further provide a strong rationale for CPB in CTCL/PTCL. While few of these patients have been included in early phase clinical trials and further experience with CPB in CTCL/PTCL is needed, few durable responses have been observed to date. Twenty-three CTCL/PTCL patients were enrolled in a phase Ib study with nivolumab in relapsed/refractory hematologic malignancies [13]. Among heavily pretreated (61% had received ≥4 prior therapies) CTCL/PTCL patients enrolled in this study, no complete remissions and 4 partial remissions were observed, for an overall response rate of 17% [13]. While the median progression-free survival was 10 weeks for all patients, two responding CTCL patients achieved responses that were ongoing at 24+ and 50+ weeks. A single PTCL patient achieved a response that was ongoing at 18+ months. Preliminary data from an ongoing phase II study with pembrolizumab in relapsed/refractory mycosis fungoides (MF) and Sezary syndrome (SS) has been reported [27]. Among 24 patients enrolled, no complete remissions and eight partial remissions were observed, for an overall response rate (ORR) of 33%. Among these responses, four were in MF (44% ORR in MF) and four in Sezary syndrome (27% ORR in SS). Responses were observed in advanced-stage MF, including patients with tumor-stage disease (2/2, ORR 100%) and large-cell transformation (1/3, ORR 33%). While these preliminary results are encouraging, improved understanding of the genomic and immunologic landscapes may be needed to further optimize CPB in the T-cell lymphoproliferative disorders.

### Challenges to checkpoint blockade in the T-cell lymphoproliferative disorders

#### Genomic complexity and neoantigen load

In addition to PD-L1 expression itself, the burden of nonsynonymous mutations and neoantigens has emerged as an important biomarker in CPB-treated patients. The frequency of mutations is highly variable across tumor types (and within a given tumor type). Carcinogen-associated tumors, most notably melanoma and non-small cell lung cancer (NSCLC), are associated with both a relatively high frequency of somatic mutations (≈10/Mb) and superior response rates to CPB [28] that is likely explained by immune-mediated destruction of neoantigen-expressing tumors [29–32]. For example, in melanoma patients treated with CTLA-4 CPB, a high mutational load was associated with clinical benefit from CPB [31, 32]. The overwhelming majority of patients who derived clinical benefit from CPB had >100 missense mutations, whereas patients who failed to benefit had a significantly lower mutational burden. A similar association between mutational burden and response to PD-1 CPB has been observed in NSCLC [29]. Despite the highly significant association between mutational and neoantigen load and response to CPB, this relationship is not absolute.

In contrast to melanoma and NSCLC, most hematologic malignancies (e.g. acute myelogenous leukemia, chronic lymphocytic leukemia, multiple myeloma) are associated with a lower frequency of somatic mutations (≤1/Mb) [28]. Recently performed next-generation sequencing studies highlight the genomic complexity associated with many T-cell NHL. Somatic copy number variants (SCNV), many of which are focal deletions/amplifications, and novel fusion events, are common in CTCL [33–35]. Catastrophic genomic rearrangements in which a chromosomal region(s) is subject to multiple double-stranded DNA breaks followed by random reassembly to form a complex mosaic chromosome, while infrequently observed in most cancers [36, 37], are highly prevalent in CTCL [33]. These studies have also implicated ultraviolet B (UVB) radiation in CTCL pathogenesis [33–35, 38, 39], as a high frequency of C > T transitions have been observed, in contrast to the T > G transversions observed in B-cell lymphproliferative disorders [40]. Many of these occurred at NpCpC trinucleotides, a signature associated with UVB exposure in melanoma [40]. In addition to this global genomic complexity, nonsynonymous point mutations occur in CTCL at a rate of ≈ 3 mutations/Mb, a rate that is substantially higher than many other hematologic malignancies [28]. An average of ≈ 50–100 somatic nonsynonymous mutations are observed in CTCL, but considerable variability with mutation rates exceeding 300/tumor in some cases have been appreciated [33–35, 38, 39, 41]. By comparison, the mutation rate (≈25/tumor) appears to be lower in PTCL [42, 43]. While not uniformly reported, these studies suggest that the mutational load varies by histology and disease stage. For example, a nonsynonymous mutation rate exceeding 5/Mb was observed in 29% (5/17) of CTCL tumors that had undergone large-cell transformation [38]. This mutation rate exceeds that observed in most lymphoproliferative disorders, and approaches that observed in many melanomas [28]. Therefore, distinct subsets of T-cell NHL likely possess a mutational burden that rivals that associated with clinical benefit from CPB in solid tumors like melanoma and NSCLC.

In addition to mutational burden and the presentation of clonal neoantigens [30], specific mutations, including those that are prevalent in selected T-cell NHL, may confer susceptibility to CPB. In an effort to understand the mechanisms involved in acquired resistance to CPB, Zaretsky et. al. performed whole-exome sequencing in paired initial and relapsing biopsies from four melanoma patients. Loss-of-function mutations in JAK1 and JAK2, associated with diminished responsiveness to interferon-γ induced PD-L1 expression, were observed in two patients [44]. Conversely, amplification of the JAK2 locus and enhanced JAK/STAT signaling promotes PD-L1 expression in selected B-cell malignancies [6, 45]. Therefore, gain-of-function mutations and other genetic alterations that promote JAK/STAT signaling in selected PTCL subtypes may confer susceptibility to CPB [reviewed in [46]]. Of course, the incorporation of genomic data in future and ongoing CPB trials will be required to address this question and may improve the ability to identify “exceptional responders”.

In contrast to other tumor types, including the B-cell lymphomas, the T-cell lymphomas are derived from the very cell types (e.g. effector T cells) that are required for CPB-mediated tumor eradication. Consequently, when considering CPB within this context, “the world turned upside down”. For example, recurrent mutations in both the CD28 extracellular and intracytoplasmic domains have been observed in a minority of PTCL [42, 47], and more recently in MF/SS [33, 38]. Mutations in the extracellular domain increase the binding affinity to CD28 ligands, particularly CD86 [33]. Whether CTLA-4 blockade, by increasing the availability of CD28 ligands within the tumor microenvironment, may promote the growth of malignant T cells harboring these rare mutations is unknown. Similarly, a rare in-frame fusion between the extracellular and transmembrane domain of CTLA-4 and the intracytoplasmic domain of CD28 has been observed, and presumably exploits the high-affinity ligand-binding domain of CTLA-4 to activate CD28 signaling [38, 39, 48]. A rapid, transient response was observed in one patient harboring this novel translocation following therapy with ipilimumab [48]. While PD-1 is highly expressed is many CTCL and T_FH_-derived PTCL [25, 49], it is also recurrently deleted in a subset of CTCL [38], but the implications of these observations for PD-1 CPB are uncertain.

#### The T-cell repertoire

While varying degrees of disease- and treatment-related immune suppression are observed in most hematologic malignancies (after all, these are cancers derived from immune cells that reside in primary and/or secondary lymphoid organs). Immune suppression observed in patients with advanced-stage T-cell lymphomas may be profound. In fact, infectious complications, including opportunistic infections, are a common cause of death in patients with advanced-stage CTCL [26]. In conjunction with age-related immune senescence, malignant T cells may compete with their conventional counterparts. Among patients with CTCL, particularly patients with Sezary syndrome, malignant T cells win this homeostatic tug-of-war, as the normal T-cell repertoire is severely restricted, resembling that observed in HIV [50], and thymic output dimished [51]. However, anecdotal evidence suggests that T-cell diversity may be restored among patients who achieve a remission with lymphoma-directed therapies [52]. This loss of T-cell diversity and a restricted T-cell repertoire may pose a significant challenge for CPB in selected patients. However, high-dose chemotherapy and autologous stem-cell transplantation (HDT-ASCT) is frequently employed as a consolidation strategy among patients with the more common peripheral T-cell lymphomas who achieve a first remission. While the T-cell repertoire following HDT-ASCT has not been scrutinized in these patients, the post-transplant period may be an opportune time for CPB given the improved effector to tumor cell ratio achieved post-transplant.

#### The tumor microenvironment (TME)

In contrast to most solid tumors, the “tumor” microenvironment for most T-cell lymphomas includes secondary lymphoid organs. The architectural effacement of involved lymph nodes undoubtedly contributes to the suppression of anti-tumor immunity and global immune suppression. Fibroblastic reticular cells (FRC) not only provide the structural scaffolding required for a functionally competent lymph node, but they also produce chemokine gradients (e.g. CCL19, CCL21) and homeostatic cytokines (e.g. IL-7) that are required for proper choreography of the adaptive immune response and homeostatic survival of naïve T cells [53]. Emerging evidence suggests that tumor-induced changes in the FRC network contribute to immune suppression [54], and may pose a challenge to CPB in lymphoproliferative disorders that are associated with widespread nodal involvement.

In addition to a disordered lymph node microenvironment, distinct T-cell lymphoma subsets are derived from populations of “regulatory” T cells that normally function to restrain T-cell immunity and hamper tumor-specific immunity [e.g. FoxP3^+^ T_reg_ cells, [26, 55, 56]]. More recently, a significant proportion of PTCL, NOS were shown to highly express the Th2-associated transcription factor GATA-3 and Th2-associated cytokines, both of which may contribute to the expansion and functional polarization of immunosuppressive lymphoma-associated macrophages [22, 23]. Similarly, a subset of PTCL (i.e. angioimmunoblastic T-cell lymphomas) express the transcriptional repressor Bcl-6 and are T_FH_-cell derived. These T_FH_-derived lymphomas are associated with an expanded population of B-cell immunoblasts. The extent to which these B cells have regulatory properties is poorly understood [57]. Therefore, in addition to structural alterations in secondary lymphoid organs, the “cell of origin” from which a malignant T cell is derived dramatically alters the TME in a manner that is likely relevant for CPB and other immunomodulatory therapies [46].

In addition to those characteristics of the TME that are likely most relevant for the lymphoproliferative disorders generally, and the T-cell lymphomas specifically, the TME in most T-cell lymphomas, like many solid tumors, includes expanded populations of myeloid-derived cells, most notably lymphoma-associated macrophages (LAM) and eosinophils. As in other tumor types, LAM impair tumor-specific immunity in the T-cell lymphomas, in part, by their expression of PD-L1 [21]. While the role of lymphoma-associated eosinophils (LAE) in PTCL is poorly understood, their shear abundance in many of these lymphomas coupled with their ability to suppress T-cell immunity in other contexts suggests that LAE may be relevant players within the TME and may impede CPB [58–60]. In addition to its role in suppressing cytotoxic T-cell-mediated cytotoxicity, PD-L1 promotes the generation of induced T_regs_ within the TME [21]. And, of course, constituents of the TME undoubtedly provide additional co-inhibitory ligands that engage additional immune checkpoints that are not yet characterized in T-cell lymphoproliferative disorders [61]. Given these challenges imposed by the TME, it seems quite likely that CPB as a single therapeutic modality may be further optimized by the incorporation of complementary therapeutic strategies that may overcome the barriers to effective tumor immunity following CPB alone in the T-cell lymphomas.

### Combination strategies for optimizing checkpoint blockade

#### Immunomodulatory agents

Lenalidomide is an immunomodulatory derivative (IMiD) with pleiotropic immunomodulatory effects and clinical activity in many hematologic malignancies. Lenalidomide promotes natural killer (NK) cell function [62, 63], stimulates T-cell proliferation and IL-2 production [64], promotes Th1-biased immunity [65], abrogates PD-L1 induced inhibition of NK and T-cell functions [66], and may thus augment responses observed with checkpoint blockade [67]. Multiple co-inhibitory ligands collaborate to impair formation of an organized immunologic synapse between malignant lymphocytes and effector T cells [66]. Impaired formation of the immunologic synapse is abrogated by lenalidomide [66]. The immunomodulatory effects observed in T cells exposed to lenalidomide are likely mediated by cereblon-dependent proteasomal degradation of Ikaros family transcription factors [64]. Interleukin-10 is secreted by malignant T cells and promotes immune evasion in PTCL [22, 68], and its expression in CD4^+^ T cells is positively regulated by Ikaros [69], likely explaining the mechanism by which lenalidomide impairs IL-10 production in malignant T cells (unpublished observation). A multicenter phase 2 study in patients with relapsed/refractory PTCL (*n* = 54) treated with single-agent lenalidomide demonstrated an overall response rate of 22% (11% CR). Stable disease was observed in 30%, for an overall tumor control rate of 52% [70]. Therefore, lenalidomide and CPB may be rationally combined in T-cell NHL.

Recurrent mutations or deletions in chromatin remodeling proteins (e.g. SWI/SNF complex) or histone (e.g. deacetylases) and DNA (e.g. methyltransferases) modifying enzymes are commonly observed in many T-cell NHL. Alterations in these epigenetic modifiers have been observed in >90% of CTCL and may explain, at least in part, the clinical activity associated with histone deacetylase inhibitors (HDACi) in these lymphomas [34]. Three HDACi are currently FDA approved for use in various T-cell NHL, with overall response rates from 25–30% being observed [71–73]. Histone deacetylases, by epigenetically regulating gene expression, regulate the differentiation, activation and effector functions of conventional lymphocytes [74]. Not surprisingly then, HDAC inhibitors have pleiotropic immunomodulatory effects. While the mechansims and specific HDAC’s involved are poorly understood, HDAC inhibition may promote tumor antigen presentation and T-cell costimulation [75–78], conferring increased susceptibility to cell-mediated cytotoxicity [79]. These observations are relevant, as loss of antigen presentation may be an important mechanism of acquired resistance to CPB [44]. Various HDAC inhibitors have been shown to either promote, or in some cases suppress, the function of conventional and regulatory T cells and myeloid-derived cells [reviewed in [80]]. These effects are likely dependent upon the agent used and the specificity for its targets at the dose and schedule utilized. Nonetheless, Zheng et. al. recently demonstrated using a murine NSCLC model that romidepsin significantly increased the production of chemokines required for T-cell trafficking [81]. Therefore, when combined with CPB, romidepsin led to a significant increase in tumor-infiltrating lymphocytes and improved disease control in this model. A similar mechanism was recently invoked in an ovarian cancer model [82]. In this model, epigenetic silencing of the same chemokines examined by Zheng et. al. (CXCL9, CXCL10) was associated with poor outcomes with CPB. However, treatment with a hypomethylating agent reversed this epigenetic silencing, increased migration of effector T cells to the tumor site, and significantly improved the efficacy of CPB [82]. These insights, combined with the now well-established activity and favorable toxicity profile of the HDAC inhibitors in clinical use, provide a strong rationale for combination strategies incorporating these agents.

Maturation of tumor-associated dendritic cells (DC) is required for trafficking to tumor-draining lymph nodes and optimal antigen-presentation, but is often impaired within the TME. Despite a robust infiltrate of DC in many T-cell NHL, their maturation is impaired in an IL-10-dependent manner [68]. Therefore, strategies that promote DC maturation within the TME may promote anti-tumor immunity. DC maturation is regulated by receptors, including toll-like receptors (TLR), that sense a broad spectrum of “danger signals”. For example, TLR8 is expressed by DCs within human skin and promotes their activation in response to viral ssRNA [83]. Resiquimod, an imidazoquinoline that is a potent TLR8 (and TLR7) agonist, is available as a topical gel and was investigated in patients with limited-stage CTCL [84]. In this study, resiquimod was topically applied to 4–5 target lesions in 12 patients. Complete resolution of all *treated* lesions was observed in 30% of patients and was associated with DC maturation and the recruitment of activated T cells. More impressively, this therapy was associated with complete resolution of all disease, including *untreated* lesions, in two patients. A similar strategy has been employed with an intratumorally injected TLR9 agonist combined with radiation [85]. These observations suggest that CPB may be improved by strategies that augment innate immunity and bolster antigen presentation, leading to more effective priming of antigen-specific T cells. As the skin is a commonly involved extranodal site of disease in many PTCL, these findings may have therapeutic applications, in combination with CPB, that extend beyond CTCL.

#### Targeted agents

Novel agents targeting a number of tyrosine kinases, including those that are required for antigen-receptor signaling in malignant lymphocytes, are increasingly being utilized, and are now FDA approved, in various hematologic malignancies. The role of these novel agents, and their targets, in the T-cell lymphoproliferative disorders is poorly understood, but is the subject of ongoing pre-clinical and clinical studies [46, 86]. More recently, we observed that TCR activation in primary T-cell lymphoma cells had a profound effect on gene expression, as ≈ 1000 genes were significantly upregulated following TCR engagement, including those that regulate cell growth and survival [87]. Engagement of the TCR by either antibody cross-linking or major histocompatibility complex proteins expressed by autologous lymphoma-associated macrophages increased T-cell lymphoma proliferation and increased resistance to chemotherapeutic agents in primary specimens. The Tec family kinase, and BTK homologue, ITK plays a pivotal role in proximal TCR signaling [reviewed in [88]]. Given their high homology, it may not be surprising that the BTK inhibitor ibrutinib also inhibits ITK in normal T cells [89]. Therefore, ITK inhibition has emerged as a novel therapeutic strategy in the T-cell NHL, and is the subject of an ongoing clinical trial. However, in addition to its direct effect on malignant T cells, ITK inhibition may also have relevant immunomodulatory effects, as ITK qualitatively regulates TCR signaling and negatively regulates the expression of T-bet, and is thus required for optimal Th2-biased immunity [90]. Consequently, established Th2 cells are increasingly dependent upon ITK, as the Th2-associated transcription factor GATA-3 promotes the selective downregulation of RLK, a TEC family kinase that is, at least partially, redundant with ITK. Therefore, in contrast to Th1 cells, that are associated with effective anti-tumor immunity, Th2 cells, due to a relative abundance of ITK compared with RLK (i.e. a high ITK/RLK ratio), are particularly sensitive to ibrutinib, which does not inhibit RLK. Therefore, ibrutinib therapy in murine models is associated with enhanced Th1-biased immunity [89], and augments CPB [91].

Tumor-antigen release and presentation is a prerequisite for adaptive T-cell immunity and the emergence of T-cell anergy or exhaustion. Therefore, T-cell “ignorance” may be fostered by non-immunogenic forms of cell death in some tumors [92, 93], and pose a barrier to effective CPB. In contrast, immunogenic forms of cell death (ICD) are associated with the unveiling of various “danger signals” that foster antigen uptake, presentation, and the induction of T-cell immunity [94]. A number of “conventional” therapies, including selected chemotherapeutic agents and radiation [95, 96], are potent inducers of ICD, and may augment CPB if properly sequenced. For example, radiation of a single tumor may initiate an anti-tumor immune response, culminating in the eradication of distant tumor deposits when used in conjunction with CPB [97, 98]. While definitive radiation therapy is used infrequently in most T-cell NHL, palliative radiation therapy is frequently utilized in tumor-stage CTCL. Tumor cell autophagy (“self-eating”) promotes the release of “danger signals” and tumor antigens required for ICD. Therefore, impaired autophagy abrogates chemotherapy-induced ICD and anti-tumor immunity, while the induction of autophagy promotes ICD [99, 100]. Therapeutic strategies that induce autophagy may be rationally combined with CPB. The mammalian target of rapamycin (mTOR), in response to nutritional and environmental cues, regulates a number of growth and survival pathways in cancer, including T-cell NHL [23, 101], and negatively regulates autophagy [102]. Responses following mTOR inhibition have been observed in patients with relapsed/refractory T-cell NHL, and by promoting autophagy and ICD may be rationally combined with both HDAC inhibitors and CPB [101, 103–105].

#### Targeting the tumor microenvironment

Gene expression by lymphoma-associated macrophages (LAM) and their density within the TME explains the variable natural history associated with many NHL [3]. LAM are abundant constituents of the TME in PTCL, where they promote chemotherapy resistance and suppress anti-tumor immunity [22, 46, 68]. Upon activation and differentiation, normal CD4^+^ T cells produce cytokines that regulate LAM biology, including type 1 (IFN-γ) and type 2 (IL-4, IL-13) cytokines, which lead to “classical” and “alternative” pathways of LAM activation and functional polarization, respectively. Cytokines produced by malignant T cells similarly promote LAM recruitment/expansion within the TME [68], their functional (“alternative”) polarization [22], and PD-L1 expression [21]. Thus, depletion of LAM in the T-cell lymphomas may not only impair the growth and survival of malignant T cells, but reverse immune suppression within the TME. While a number of tumor-derived factors have been implicated in the recruitment and survival of LAM, colony-stimulating factor-1 (CSF-1, or M-CSF) is required for normal macrophage homeostasis and viability. Mice lacking functional CSF-1 or nullizygous for CSF-1 receptor (CSF-1R, c-fms, CD115) have a marked decrease in tissue resident macrophages. More importantly, CSF-1R blockade prevents the generation of LAM in a variety of hematologic malignancies [106]. Therefore, inhibition of CSF-1R has emerged as a rational therapeutic strategy in many solid tumors and hematologic malignancies [107–111]. Therefore, depletion of LAM from the TME may augment the efficacy of novel immunotherapies, including CPB [112]. Second only to LAM, eosinophils are arguably an abundant subset of myeloid-derived cells in many T-cell NHL. While their role in these lymphomas is poorly understood, eosinophils are an abundant source of indoleamine 2,3 dioxygenase (IDO) [58], the rate-limiting enzyme in catabolism of the essential amino acid tryptophan. As tryptophan is required for optimal T-cell immunity, IDO is an alternative “checkpoint” within the TME that may confer resistance to CPB [113].

The chemokine receptor CCR4 is preferentially expressed by Th2 and T_reg_ cells and promotes their migration to extranodal sites, including the skin, in response to its ligands (CCL17, CCL22). CCR4 is widely expressed in CTCL and a recently described subset of PTCL, NOS [22]. The CCL17/CCL22 - CCR4 axis in CTCL is maintained by the absence of CD26, a dipeptydilpeptidase, which inactivates CCR4 ligands. Recently described gain-of-function mutations in the CCR4 cytoplasmic domain inhibit CCR4 internalization and promote PI3K/AKT activation. These mutations were recently described in ≈ 25% of adult T-cell leukemia/lymphomas and in CTCL [38, 114]. Therefore, CCR4 has a pathogenic role in selected T-cell NHL and has been the subject of ongoing investigations with the anti-CCR4 monoclonal antibody mogamulizumab. This glycoengineered monoclonal antibody depletes CCR4-expressing cells by antibody-dependent cell-mediated cytotoxicity (ADCC), and has significant clinical activity in CCR4^+^ T-cell NHL [115–117]. However, in addition to directly targeting malignant T cells, mogamulizumab depletes T_regs_ in these patients [116, 118] Therefore, mogamulizumab, by depleting malignant T cells and T_regs_, effectively kills “two birds with one stone” in the T-cell NHL [119]. Not surprisingly, T_reg_ depletion with this agent has been associated with improved anti-tumor immunity [120, 121], and future studies in combination with CPB in the T-cell NHL are warranted.

## Conclusions

Conventional chemotherapeutic strategies are not curative for the majority of patients with T-cell lymphoproliferative disorders. This may be explained by both cell-autonomous and non-cell-autonomous mechanisms of chemotherapy resistance. Prior to the dawn of the new era in immunotherapy and immune checkpoint blockade, chemotherapy was similarly ineffective in metastatic melanoma, but durable remissions, if not cure, are now achieved with checkpoint blockade in this disease. Improved understanding of the genetic landscape in the T-cell lymphomas and the use of novel targeted agents, while welcome additions to the therapeutic armamentarium, are unlikely to be curative for most patients with these aggressive lymphomas. In contrast, further optimization of checkpoint blockade, and other immunotherapeutic strategies, combined with kinase inhibitors, immunomodulatory agents, or therapies targeting the epigenome offer the hope that conventional multi-agent chemotherapy may be replaced by well tolerated therapies with curative potential for patients afflicted with these aggressive lymphomas.

## Abbreviations

ADCC: Antibody-dependent cell-mediated cytotoxicity
ALCL: Anaplastic large cell lymphomas
ATLL: Adult T-cell leukemia/lymphomas
CPB: Checkpoint blockade
CR: Complete response
CSF: Colony-stimulating factor
CTCL: Cutaneous T-cell lymphoma
DC: Dendritic cells
ECP: Extracorporeal photopheresis
FRC: Fibroblastic reticular cells
HDAC: Histone deacetylase
ICD: Immunogenic forms of cell death
IMiD: Immunomodulatory derivative
LAE: Lymphoma-associated eosinophils
LAM: Lymphoma-associated macrophages
Mb: Megabase
MF: Mycosis fungoides
mTOR: Mammalian target of rapamycin
NHL: Non-Hodgkin’s lymphoma
NK: Natural killer
NOS: Not otherwise specified
NSCLC: Non-small cell lung cancer
ORR: Overall response rate
PR: Partial response
PTCL: Peripheral T-cell lymphoma
SCNV: Somatic copy number variants
SS: Sezary syndrome
SWI/SNF: SWItch/sucrose non-fermentable
TCR: T-cell receptor
TLR: Toll-like receptor
TME: Tumor microenvironment
UVB: Ultraviolet B

## References

[1] Postow MA, Callahan MK, Wolchok JD (2015). Immune Checkpoint Blockade in Cancer Therapy. J Clin Oncol.

[2] Scott DW, Gascoyne RD (2014). The tumour microenvironment in B cell lymphomas. Nat Rev Cancer.

[3] Dave SS, Wright G, Tan B, Rosenwald A, Gascoyne RD, Chan WC, Fisher RI, Braziel RM, Rimsza LM, Grogan TM (2004). Prediction of survival in follicular lymphoma based on molecular features of tumor-infiltrating immune cells. N Engl J Med.

[4] Georgiou K, Chen L, Berglund M, Ren W, de Miranda NF, Lisboa S, Fangazio M, Zhu S, Hou Y, Wu K (2016). Genetic basis of PD-L1 overexpression in diffuse large B-cell lymphomas. Blood.

[5] Keane C, Vari F, Hertzberg M, Cao KA, Green MR, Han E, Seymour JF, Hicks RJ, Gill D, Crooks P (2015). Ratios of T-cell immune effectors and checkpoint molecules as prognostic biomarkers in diffuse large B-cell lymphoma: a population-based study. Lancet Haematol.

[6] Green MR, Monti S, Rodig SJ, Juszczynski P, Currie T, O’Donnell E, Chapuy B, Takeyama K, Neuberg D, Golub TR (2010). Integrative analysis reveals selective 9p24.1 amplification, increased PD-1 ligand expression, and further induction via JAK2 in nodular sclerosing Hodgkin lymphoma and primary mediastinal large B-cell lymphoma. Blood.

[7] Blaker YN, Spetalen S, Brodtkorb M, Lingjaerde OC, Beiske K, Ostenstad B, Sander B, Wahlin BE, Melen CM, Myklebust JH, et al. The tumour microenvironment influences survival and time to transformation in follicular lymphoma in the rituximab era. Br J Haematol. 2016. 10.1111/bjh.14201 27341313

[8] Kiyasu J, Miyoshi H, Hirata A, Arakawa F, Ichikawa A, Niino D, Sugita Y, Yufu Y, Choi I, Abe Y (2015). Expression of programmed cell death ligand 1 is associated with poor overall survival in patients with diffuse large B-cell lymphoma. Blood.

[9] Younes A, Santoro A, Shipp M, Zinzani PL, Timmerman JM, Ansell S, Armand P, Fanale M, Ratanatharathorn V, Kuruvilla J, et al. Nivolumab for classical Hodgkin’s lymphoma after failure of both autologous stem-cell transplantation and brentuximab vedotin: a multicentre, multicohort, single-arm phase 2 trial. Lancet Oncol. 2016. 10.1016/S1470-2045(16)30167-X PMC5541855 27451390

[10] Ansell SM, Lesokhin AM, Borrello I, Halwani A, Scott EC, Gutierrez M, Schuster SJ, Millenson MM, Cattry D, Freeman GJ (2015). PD-1 blockade with nivolumab in relapsed or refractory Hodgkin’s lymphoma. N Engl J Med.

[11] Armand P, Shipp MA, Ribrag V, Michot JM, Zinzani PL, Kuruvilla J, Snyder ES, Ricart AD, Balakumaran A, Rose S, Moskowitz CH. Programmed Death-1 Blockade With Pembrolizumab in Patients With Classical Hodgkin Lymphoma After Brentuximab Vedotin Failure. J Clin Oncol. 2016. 10.1200/JCO.2016.67.3467 PMC5791838 27354476

[12] Westin JR, Chu F, Zhang M, Fayad LE, Kwak LW, Fowler N, Romaguera J, Hagemeister F, Fanale M, Samaniego F (2014). Safety and activity of PD1 blockade by pidilizumab in combination with rituximab in patients with relapsed follicular lymphoma: a single group, open-label, phase 2 trial. Lancet Oncol.

[13] Lesokhin AM, Ansell SM, Armand P, Scott EC, Halwani A, Gutierrez M, Millenson MM, Cohen AD, Schuster SJ, Lebovic D, et al. Nivolumab in Patients With Relapsed or Refractory Hematologic Malignancy: Preliminary Results of a Phase Ib Study. J Clin Oncol. 2016. 10.1200/JCO.2015.65.9789 PMC5019749 27269947

[14] Armand P, Nagler A, Weller EA, Devine SM, Avigan DE, Chen YB, Kaminski MS, Holland HK, Winter JN, Mason JR (2013). Disabling immune tolerance by programmed death-1 blockade with pidilizumab after autologous hematopoietic stem-cell transplantation for diffuse large B-cell lymphoma: results of an international phase II trial. J Clin Oncol.

[15] Vose J, Armitage J, Weisenburger D (2008). International peripheral T-cell and natural killer/T-cell lymphoma study: pathology findings and clinical outcomes. J Clin Oncol.

[16] Briski R, Feldman AL, Bailey NG, Lim MS, Ristow K, Habermann TM, Macon WR, Inwards DJ, Colgan JP, Nowakowski GS (2014). The role of front-line anthracycline-containing chemotherapy regimens in peripheral T-cell lymphomas. Blood Cancer J.

[17] Mak V, Hamm J, Chhanabhai M, Shenkier T, Klasa R, Sehn LH, Villa D, Gascoyne RD, Connors JM, Savage KJ (2013). Survival of patients with peripheral T-cell lymphoma after first relapse or progression: spectrum of disease and rare long-term survivors. J Clin Oncol.

[18] Briski R, Feldman AL, Bailey NG, Lim MS, Ristow K, Habermann TM, Macon WR, Inwards DJ, Colgan JP, Nowakowski GS, et al. Survival in Patients with Limited-stage Peripheral T-cell Lymphomas. Leuk Lymphoma. 2014;56:1-17. 10.3109/10428194.2014.963078 PMC4456336 25248884

[19] Hughes CF, Khot A, McCormack C, Lade S, Westerman DA, Twigger R, Buelens O, Newland K, Tam C, Dickinson M (2015). Lack of durable disease control with chemotherapy for mycosis fungoides and Sezary syndrome: a comparative study of systemic therapy. Blood.

[20] Agar NS, Wedgeworth E, Crichton S, Mitchell TJ, Cox M, Ferreira S, Robson A, Calonje E, Stefanato CM, Wain EM (2010). Survival outcomes and prognostic factors in mycosis fungoides/Sezary syndrome: validation of the revised International Society for Cutaneous Lymphomas/European Organisation for Research and Treatment of Cancer staging proposal. J Clin Oncol.

[21] Wilcox RA, Feldman AL, Wada DA, Yang ZZ, Comfere NI, Dong H, Kwon ED, Novak AJ, Markovic SN, Pittelkow MR (2009). B7-H1 (PD-L1, CD274) suppresses host immunity in T-cell lymphoproliferative disorders. Blood.

[22] Wang T, Feldman AL, Wada DA, Lu Y, Polk A, Briski R, Ristow K, Habermann TM, Thomas D, Ziesmer SC (2014). GATA-3 expression identifies a high-risk subset of PTCL, NOS with distinct molecular and clinical features. Blood.

[23] Iqbal J, Wright G, Wang C, Rosenwald A, Gascoyne RD, Weisenburger DD, Greiner TC, Smith L, Guo S, Wilcox RA (2014). Gene expression signatures delineate biological and prognostic subgroups in peripheral T-cell lymphoma. Blood.

[24] Kataoka K, Shiraishi Y, Takeda Y, Sakata S, Matsumoto M, Nagano S, Maeda T, Nagata Y, Kitanaka A, Mizuno S (2016). Aberrant PD-L1 expression through 3′-UTR disruption in multiple cancers. Nature.

[25] Wilcox RA, Ansell SM, Lim MS, Zou W, Chen L (2012). The B7 homologues and their receptors in hematologic malignancies. Eur J Haematol.

[26] Wilcox RA (2011). Cutaneous T-cell lymphoma: 2011 update on diagnosis, risk-stratification, and management. Am J Hematol.

[27] Kim Y. A Phase 2 Study of Pembrolizumab for the Treatment of Relapsed/Refractory MF/SS. Presented at: T-cell Lymphoma Forum 2016

[28] Lawrence MS, Stojanov P, Mermel CH, Robinson JT, Garraway LA, Golub TR, Meyerson M, Gabriel SB, Lander ES, Getz G (2014). Discovery and saturation analysis of cancer genes across 21 tumour types. Nature.

[29] Rizvi NA, Hellmann MD, Snyder A, Kvistborg P, Makarov V, Havel JJ, Lee W, Yuan J, Wong P, Ho TS (2015). Cancer immunology. Mutational landscape determines sensitivity to PD-1 blockade in non-small cell lung cancer. Science.

[30] McGranahan N, Furness AJ, Rosenthal R, Ramskov S, Lyngaa R, Saini SK, Jamal-Hanjani M, Wilson GA, Birkbak NJ, Hiley CT (2016). Clonal neoantigens elicit T cell immunoreactivity and sensitivity to immune checkpoint blockade. Science.

[31] Van Allen EM, Miao D, Schilling B, Shukla SA, Blank C, Zimmer L, Sucker A, Hillen U, Geukes Foppen MH, Goldinger SM (2015). Genomic correlates of response to CTLA-4 blockade in metastatic melanoma. Science.

[32] Snyder A, Makarov V, Merghoub T, Yuan J, Zaretsky JM, Desrichard A, Walsh LA, Postow MA, Wong P, Ho TS (2014). Genetic basis for clinical response to CTLA-4 blockade in melanoma. N Engl J Med.

[33] Choi J, Goh G, Walradt T, Hong BS, Bunick CG, Chen K, Bjornson RD, Maman Y, Wang T, Tordoff J (2015). Genomic landscape of cutaneous T cell lymphoma. Nat Genet.

[34] Kiel MJ, Sahasrabuddhe AA, Rolland DC, Velusamy T, Chung F, Schaller M, Bailey NG, Betz BL, Miranda RN, Porcu P (2015). Genomic analyses reveal recurrent mutations in epigenetic modifiers and the JAK-STAT pathway in Sezary syndrome. Nat Commun.

[35] da Silva Almeida AC, Abate F, Khiabanian H, Martinez-Escala E, Guitart J, Tensen CP, Vermeer MH, Rabadan R, Ferrando A, Palomero T (2015). The mutational landscape of cutaneous T cell lymphoma and Sezary syndrome. Nat Genet.

[36] Kloosterman WP, Koster J, Molenaar JJ (2014). Prevalence and clinical implications of chromothripsis in cancer genomes. Curr Opin Oncol.

[37] Zack TI, Schumacher SE, Carter SL, Cherniack AD, Saksena G, Tabak B, Lawrence MS, Zhsng CZ, Wala J, Mermel CH (2013). Pan-cancer patterns of somatic copy number alteration. Nat Genet.

[38] Wang L, Ni X, Covington KR, Yang BY, Shiu J, Zhang X, Xi L, Meng Q, Langridge T, Drummond J (2015). Genomic profiling of Sezary syndrome identifies alterations of key T cell signaling and differentiation genes. Nat Genet.

[39] Ungewickell A, Bhaduri A, Rios E, Reuter J, Lee CS, Mah A, Zehnder A, Ohgami R, Kulkarni S, Armstrong R (2015). Genomic analysis of mycosis fungoides and Sezary syndrome identifies recurrent alterations in TNFR2. Nat Genet.

[40] Alexandrov LB, Nik-Zainal S, Wedge DC, Aparicio SA, Behjati S, Biankin AV, Bignell GR, Bolli N, Borg A, Borresen-Dale AL (2013). Signatures of mutational processes in human cancer. Nature.

[41] Woollard WJ, Pullabhatla V, Lorenc A, Patel VM, Butler RM, Bayega A, Begum N, Bakr F, Dedhia K, Fisher J (2016). Candidate driver genes involved in genome maintenance and DNA repair in Sezary syndrome. Blood.

[42] Yoo HY, Sung MK, Lee SH, Kim S, Lee H, Park S, Kim SC, Lee B, Rho K, Lee JE (2014). A recurrent inactivating mutation in RHOA GTPase in angioimmunoblastic T cell lymphoma. Nat Genet.

[43] Palomero T, Couronne L, Khiabanian H, Kim MY, Ambesi-Impiombato A, Perez-Garcia A, Carpenter Z, Abate F, Allegretta M, Haydu JE (2014). Recurrent mutations in epigenetic regulators, RHOA and FYN kinase in peripheral T cell lymphomas. Nat Genet.

[44] Zaretsky JM, Garcia-Diaz A, Shin DS, Escuin-Ordinas H, Hugo W, Hu-Lieskovan S, Torrejon DY, Abril-Rodriguez G, Sandoval S, Barthly L, et al. Mutations Associated with Acquired Resistance to PD-1 Blockade in Melanoma. N Engl J Med. 2016. 10.1056/NEJMoa1604958 PMC5007206 27433843

[45] Green MR, Rodig S, Juszczynski P, Ouyang J, Sinha P, O’Donnell E, Neuberg D, Shipp MA (2012). Constitutive AP-1 activity and EBV infection induce PD-L1 in Hodgkin lymphomas and posttransplant lymphoproliferative disorders: implications for targeted therapy. Clin Cancer Res.

[46] Wilcox RA (2016). A three-signal model of T-cell lymphoma pathogenesis. Am J Hematol.

[47] Rohr J, Guo S, Hu D, Bouska A, Gascoyne RD, Rosenwald A, Simone P, Zhang W, Xiao W, Wang C (2014). CD28 Mutations in Peripheral T-cell Lymphomagenesis and Progression. Blood Ann Meeting Abstracts.

[48] Sekulic A, Liang WS, Tembe W, Izatt T, Kruglyak S, Kiefer JA, Cuyugan L, Zismann V, Legendre C, Pittelkow M (2015). Personalized treatment of Sezary syndrome by targeting a novel CTLA4:CD28 fusion. Mol Genet Genomic Med.

[49] Samimi S, Benoit B, Evans K, Wherry EJ, Showe L, Wysocka M, Rook AH (2010). Increased programmed death-1 expression on CD4+ T cells in cutaneous T-cell lymphoma: implications for immune suppression. Arch Dermatol.

[50] Yawalkar N, Ferenczi K, Jones DA, Yamanaka K, Suh KY, Sadat S, Kupper TS (2003). Profound loss of T-cell receptor repertoire complexity in cutaneous T-cell lymphoma. Blood.

[51] Yamanaka K, Yawalkar N, Jones DA, Hurwitz D, Ferenczi K, Eapen S, Kupper TS (2005). Decreased T-cell receptor excision circles in cutaneous T-cell lymphoma. Clin Cancer Res.

[52] Yamanaka K, Fuhlbrigge RC, Mizutani H, Kupper TS (2010). Restoration of peripheral blood T cell repertoire complexity during remission in advanced cutaneous T cell lymphoma. Arch Dermatol Res.

[53] Fletcher AL, Acton SE, Knoblich K (2015). Lymph node fibroblastic reticular cells in health and disease. Nat Rev Immunol.

[54] Riedel A, Shorthouse D, Haas L, Hall BA, Shields J. Tumor-induced stromal reprogramming drives lymph node transformation. Nat Immunol. 2016;17(9):1118–27. 10.1038/ni.3492 PMC4994871 27400148

[55] Kohno T, Yamada Y, Akamatsu N, Kamihira S, Imaizumi Y, Tomonaga M, Matsuyama T (2005). Possible origin of adult T-cell leukemia/lymphoma cells from human T lymphotropic virus type-1-infected regulatory T cells. Cancer Sci.

[56] Yano H, Ishida T, Inagaki A, Ishii T, Kusumoto S, Komatsu H, Iida S, Utsunomiya A, Ueda R (2007). Regulatory T-cell function of adult T-cell leukemia/lymphoma cells. Int J Cancer.

[57] Schwartz M, Zhang Y, Rosenblatt JD (2016). B cell regulation of the anti-tumor response and role in carcinogenesis. J Immunother Cancer.

[58] Odemuyiwa SO, Ghahary A, Li Y, Puttagunta L, Lee JE, Musat-Marcu S, Ghahary A, Moqbel R (2004). Cutting edge: human eosinophils regulate T cell subset selection through indoleamine 2,3-dioxygenase. J Immunol.

[59] Nakagome K, Dohi M, Okunishi K, Tanaka R, Kouro T, Kano MR, Miyazono K, Miyazaki J, Takatsu K, Yamamoto K (2007). IL-5-induced hypereosinophilia suppresses the antigen-induced immune response via a TGF-beta-dependent mechanism. J Immunol.

[60] Jacobsen EA, Helmers RA, Lee JJ, Lee NA (2012). The expanding role(s) of eosinophils in health and disease. Blood.

[61] Anderson AC, Joller N, Kuchroo VK (2016). Lag-3, Tim-3, and TIGIT: Co-inhibitory Receptors with Specialized Functions in Immune Regulation. Immunity.

[62] Reddy N, Hernandez-Ilizaliturri FJ, Deeb G, Roth M, Vaughn M, Knight J, Wallace P, Czuczman MS (2008). Immunomodulatory drugs stimulate natural killer-cell function, alter cytokine production by dendritic cells, and inhibit angiogenesis enhancing the anti-tumour activity of rituximab in vivo. Br J Haematol.

[63] Wu L, Adams M, Carter T, Chen R, Muller G, Stirling D, Schafer P, Bartlett JB (2008). lenalidomide enhances natural killer cell and monocyte-mediated antibody-dependent cellular cytotoxicity of rituximab-treated CD20+ tumor cells. Clin Cancer Res.

[64] Kronke J, Udeshi ND, Narla A, Grauman P, Hurst SN, McConkey M, Svinkina T, Heckl D, Comer E, Li X (2014). Lenalidomide causes selective degradation of IKZF1 and IKZF3 in multiple myeloma cells. Science.

[65] Xu W, Celeridad M, Sankar S, Webb DR, Bennett BL (2008). CC-4047 promotes Th1 cell differentiation and reprograms polarized human Th2 cells by enhancing transcription factor T-bet. Clin Immunol.

[66] Ramsay AG, Clear AJ, Fatah R, Gribben JG (2012). Multiple inhibitory ligands induce impaired T-cell immunologic synapse function in chronic lymphocytic leukemia that can be blocked with lenalidomide: establishing a reversible immune evasion mechanism in human cancer. Blood.

[67] Gorgun G, Samur MK, Cowens KB, Paula S, Bianchi G, Anderson JE, White RE, Singh A, Ohguchi H, Suzuki R (2015). Lenalidomide Enhances Immune Checkpoint Blockade-Induced Immune Response in Multiple Myeloma. Clin Cancer Res.

[68] Wilcox RA, Wada DA, Ziesmer SC, Elsawa SF, Comfere NI, Dietz AB, Novak AJ, Witzig TE, Feldman AL, Pittelkow MR, Ansell SM (2009). Monocytes promote tumor cell survival in T-cell lymphoproliferative disorders and are impaired in their ability to differentiate into mature dendritic cells. Blood.

[69] Umetsu SE, Winandy S (2009). Ikaros is a regulator of Il10 expression in CD4+ T cells. J Immunol.

[70] Morschhauser F, Fitoussi O, Haioun C, Thieblemont C, Quach H, Delarue R, Glaisner S, Gabarre J, Bosly A, Lister J (2013). A phase 2, multicentre, single-arm, open-label study to evaluate the safety and efficacy of single-agent lenalidomide (Revlimid) in subjects with relapsed or refractory peripheral T-cell non-Hodgkin lymphoma: the EXPECT trial. Eur J Cancer.

[71] Coiffier B, Pro B, Prince HM, Foss F, Sokol L, Greenwood M, Caballero D, Borchmann P, Morschhauser F, Wilhelm M (2012). Results from a pivotal, open-label, phase II study of romidepsin in relapsed or refractory peripheral T-cell lymphoma after prior systemic therapy. J Clin Oncol.

[72] Olsen EA, Kim YH, Kuzel TM, Pacheco TR, Foss FM, Parker S, Frankel SR, Chen C, Ricker JL, Arduino JM, Duvic M (2007). Phase IIb multicenter trial of vorinostat in patients with persistent, progressive, or treatment refractory cutaneous T-cell lymphoma. J Clin Oncol.

[73] O’Connor OA, Horwitz S, Masszi T, Van Hoof A, Brown P, Doorduijn J, Hess G, Jurczak W, Knoblauch P, Chawla S (2015). Belinostat in Patients With Relapsed or Refractory Peripheral T-Cell Lymphoma: Results of the Pivotal Phase II BELIEF (CLN-19) Study. J Clin Oncol.

[74] Akimova T, Beier UH, Liu Y, Wang L, Hancock WW (2012). Histone/protein deacetylases and T-cell immune responses. Blood.

[75] Vo DD, Prins RM, Begley JL, Donahue TR, Morris LF, Bruhn KW, de la Rocha P, Yang MY, Mok S, Garban HJ (2009). Enhanced antitumor activity induced by adoptive T-cell transfer and adjunctive use of the histone deacetylase inhibitor LAQ824. Cancer Res.

[76] Murakami T, Sato A, Chun NA, Hara M, Naito Y, Kobayashi Y, Kano Y, Ohtsuki M, Furukawa Y, Kobayashi E (2008). Transcriptional modulation using HDACi depsipeptide promotes immune cell-mediated tumor destruction of murine B16 melanoma. J Invest Dermatol.

[77] Maeda T, Towatari M, Kosugi H, Saito H (2000). Up-regulation of costimulatory/adhesion molecules by histone deacetylase inhibitors in acute myeloid leukemia cells. Blood.

[78] Cycon KA, Mulvaney K, Rimsza LM, Persky D, Murphy SP (2013). Histone deacetylase inhibitors activate CIITA and MHC class II antigen expression in diffuse large B-cell lymphoma. Immunology.

[79] Armeanu S, Bitzer M, Lauer UM, Venturelli S, Pathil A, Krusch M, Kaiser S, Jobst J, Smirnow I, Wagner A (2005). Natural killer cell-mediated lysis of hepatoma cells via specific induction of NKG2D ligands by the histone deacetylase inhibitor sodium valproate. Cancer Res.

[80] Shen L, Orillion A, Pili R (2016). Histone deacetylase inhibitors as immunomodulators in cancer therapeutics. Epigenomics.

[81] Zheng H, Zhao W, Yan C, Watson CC, Massengill M, Xie M, Massengill C, Noyes DR, Martinez GV, Afzal R, et al. HDAC Inhibitors Enhance T-Cell Chemokine Expression and Augment Response to PD-1 Immunotherapy in Lung Adenocarcinoma. Clin Cancer Res. 2016. 10.1158/1078-0432.CCR-15-2584 PMC4987196 26964571

[82] Peng D, Kryczek I, Nagarsheth N, Zhao L, Wei S, Wang W, Sun Y, Zhao E, Vatan L, Szeliga W (2015). Epigenetic silencing of TH1-type chemokines shapes tumour immunity and immunotherapy. Nature.

[83] Gorden KB, Gorski KS, Gibson SJ, Kedl RM, Kieper WC, Qiu X, Tomai MA, Alkan SS, Vasilakos JP (2005). Synthetic TLR agonists reveal functional differences between human TLR7 and TLR8. J Immunol.

[84] Rook AH, Gelfand JM, Wysocka M, Troxel AB, Benoit B, Surber C, Elenitsas R, Buchanan MA, Leahy DS, Watanabe R (2015). Topical resiquimod can induce disease regression and enhance T-cell effector functions in cutaneous T-cell lymphoma. Blood.

[85] Kim YH, Gratzinger D, Harrison C, Brody JD, Czerwinski DK, Ai WZ, Morales A, Abdulla F, Xing L, Navi D (2012). In situ vaccination against mycosis fungoides by intratumoral injection of a TLR9 agonist combined with radiation: a phase 1/2 study. Blood.

[86] Devata S, Wilcox RA (2016). Cutaneous T-Cell Lymphoma: A Review with a Focus on Targeted Agents. Am J Clin Dermatol.

[87] Wang T, Polk A, Lu Y, Wilcox RA (2014). T-cell Receptor Engagement Confers Resistance to Chemotherapy in T-cell Lymphoproliferative Disorders. Blood (ASH Annual Meeting Abstracts).

[88] Iqbal J, Wilcox R, Naushad H, Rohr J, Heavican TB, Wang C, Bouska A, Fu K, Chan WC, Vose JM. Genomic signatures in T-cell lymphoma: How can these improve precision in diagnosis and inform prognosis? Blood Rev. 2015;30(2):89–100. 10.1016/j.blre.2015.08.003 26319391

[89] Dubovsky JA, Beckwith KA, Natarajan G, Woyach JA, Jaglowski S, Zhong Y, Hessler JD, Liu TM, Chang BY, Larkin KM (2013). Ibrutinib is an irreversible molecular inhibitor of ITK driving a Th1-selective pressure in T lymphocytes. Blood.

[90] Miller AT, Wilcox HM, Lai Z, Berg LJ (2004). Signaling through Itk promotes T helper 2 differentiation via negative regulation of T-bet. Immunity.

[91] Sagiv-Barfi I, Kohrt HE, Czerwinski DK, Ng PP, Chang BY, Levy R (2015). Therapeutic antitumor immunity by checkpoint blockade is enhanced by ibrutinib, an inhibitor of both BTK and ITK. Proc Natl Acad Sci U S A.

[92] Wilcox RA, Flies DB, Zhu G, Johnson AJ, Tamada K, Chapoval AI, Strome SE, Pease LR, Chen L (2002). Provision of antigen and CD137 signaling breaks immunological ignorance, promoting regression of poorly immunogenic tumors. J Clin Invest.

[93] Sharma P, Allison JP (2015). Immune checkpoint targeting in cancer therapy: toward combination strategies with curative potential. Cell.

[94] Zhong Z, Sanchez-Lopez E, Karin M (2016). Autophagy, Inflammation, and Immunity: A Troika Governing Cancer and Its Treatment. Cell.

[95] Zitvogel L, Apetoh L, Ghiringhelli F, Kroemer G (2008). Immunological aspects of cancer chemotherapy. Nat Rev Immunol.

[96] Galluzzi L, Buque A, Kepp O, Zitvogel L, Kroemer G (2015). Immunological Effects of Conventional Chemotherapy and Targeted Anticancer Agents. Cancer Cell.

[97] Sharabi AB, Lim M, DeWeese TL, Drake CG (2015). Radiation and checkpoint blockade immunotherapy: radiosensitisation and potential mechanisms of synergy. Lancet Oncol.

[98] Twyman-Saint Victor C, Rech AJ, Maity A, Rengan R, Pauken KE, Stelekati E, Benci JL, Xu B, Dada H, Odorizzi PM (2015). Radiation and dual checkpoint blockade activate non-redundant immune mechanisms in cancer. Nature.

[99] Michaud M, Martins I, Sukkurwala AQ, Adjemian S, Ma Y, Pellegatti P, Shen S, Kepp O, Scoazec M, Mignot G (2011). Autophagy-dependent anticancer immune responses induced by chemotherapeutic agents in mice. Science.

[100] Martins I, Wang Y, Michaud M, Ma Y, Sukkurwala AQ, Shen S, Kepp O, Metivier D, Galluzzi L, Perfettini JL (2014). Molecular mechanisms of ATP secretion during immunogenic cell death. Cell Death Differ.

[101] Witzig TE, Reeder C, Han JJ, LaPlant B, Stenson M, Tun HW, Macon W, Ansell SM, Habermann TM, Inwards DJ (2015). The mTORC1 inhibitor everolimus has antitumor activity in vitro and produces tumor responses in patients with relapsed T-cell lymphoma. Blood.

[102] Kim YC, Guan KL (2015). mTOR: a pharmacologic target for autophagy regulation. J Clin Invest.

[103] Chapman NM, Chi H (2014). mTOR signaling, Tregs and immune modulation. Immunotherapy.

[104] Jiang Q, Weiss JM, Back T, Chan T, Ortaldo JR, Guichard S, Wiltrout RH (2011). mTOR kinase inhibitor AZD8055 enhances the immunotherapeutic activity of an agonist CD40 antibody in cancer treatment. Cancer Res.

[105] Gupta M, Ansell SM, Novak AJ, Kumar S, Kaufmann SH, Witzig TE (2009). Inhibition of histone deacetylase overcomes rapamycin-mediated resistance in diffuse large B-cell lymphoma by inhibiting Akt signaling through mTORC2. Blood.

[106] Polk A, Lu Y, Wang T, Seymour E, Bailey NG, Singer JW, Boonstra PS, Lim MS, Malek S, Wilcox RA. Colony-stimulating Factor-1 Receptor is Required for Nurse-like Cell Survival in Chronic Lymphocytic Leukemia. Clin Cancer Res. 2016. 10.1158/1078-0432.CCR-15-3099 PMC5161678 27334834

[107] Hume DA, MacDonald KP (2012). Therapeutic applications of macrophage colony-stimulating factor-1 (CSF-1) and antagonists of CSF-1 receptor (CSF-1R) signaling. Blood.

[108] Pyonteck SM, Akkari L, Schuhmacher AJ, Bowman RL, Sevenich L, Quail DF, Olson OC, Quick ML, Huse JT, Teijeiro V (2013). CSF-1R inhibition alters macrophage polarization and blocks glioma progression. Nat Med.

[109] Ries CH, Cannarile MA, Hoves S, Benz J, Wartha K, Runza V, Rey-Giraud F, Pradel LP, Feuerhake F, Klaman I (2014). Targeting tumor-associated macrophages with anti-CSF-1R antibody reveals a strategy for cancer therapy. Cancer Cell.

[110] Mok S, Koya RC, Tsui C, Xu J, Robert L, Wu L, Graeber TG, West BL, Bollag G, Ribas A (2014). Inhibition of CSF-1 receptor improves the antitumor efficacy of adoptive cell transfer immunotherapy. Cancer Res.

[111] Stafford JH, Hirai T, Deng L, Chernikova SB, Urata K, West BL, Brown JM. Colony stimulating factor 1 receptor inhibition delays recurrence of glioblastoma after radiation by altering myeloid cell recruitment and polarization. Neuro Oncol. 2015. 10.1093/neuonc/nov272 PMC4864255 26538619

[112] McClanahan F, Hanna B, Miller S, Clear AJ, Lichter P, Gribben JG, Seiffert M (2015). PD-L1 checkpoint blockade prevents immune dysfunction and leukemia development in a mouse model of chronic lymphocytic leukemia. Blood.

[113] Holmgaard RB, Zamarin D, Munn DH, Wolchok JD, Allison JP (2013). Indoleamine 2,3-dioxygenase is a critical resistance mechanism in antitumor T cell immunotherapy targeting CTLA-4. J Exp Med.

[114] Nakagawa M, Schmitz R, Xiao W, Goldman CK, Xu W, Yang Y, Yu X, Waldmann TA, Staudt LM (2014). Gain-of-function CCR4 mutations in adult T cell leukemia/lymphoma. J Exp Med.

[115] Duvic M, Pinter-Brown LC, Foss FM, Sokol L, Jorgensen JL, Challagundla P, Dwyer KM, Zhang X, Kurman MR, Ballerini R (2015). Phase 1/2 study of mogamulizumab, a defucosylated anti-CCR4 antibody, in previously treated patients with cutaneous T-cell lymphoma. Blood.

[116] Ogura M, Ishida T, Hatake K, Taniwaki M, Ando K, Tobinai K, Fujimoto K, Yamamoto K, Miyamoto T, Uike N (2014). Multicenter phase II study of mogamulizumab (KW-0761), a defucosylated anti-cc chemokine receptor 4 antibody, in patients with relapsed peripheral T-cell lymphoma and cutaneous T-cell lymphoma. J Clin Oncol.

[117] Tobinai K, Takahashi T, Akinaga S (2012). Targeting chemokine receptor CCR4 in adult T-cell leukemia-lymphoma and other T-cell lymphomas. Curr Hematol Malig Rep.

[118] Ni X, Jorgensen JL, Goswami M, Challagundla P, Decker WK, Kim YH, Duvic MA (2015). Reduction of Regulatory T Cells by Mogamulizumab, a Defucosylated Anti-CC Chemokine Receptor 4 Antibody, in Patients with Aggressive/Refractory Mycosis Fungoides and Sezary Syndrome. Clin Cancer Res.

[119] Wilcox RA (2015). Mogamulizumab: 2 birds, 1 stone. Blood.

[120] Chang DK, Peterson E, Sun J, Goudie C, Drapkin RI, Liu JF, Matulonis U, Zhu Q, Marasco WA (2016). Anti-CCR4 monoclonal antibody enhances antitumor immunity by modulating tumor-infiltrating Tregs in an ovarian cancer xenograft humanized mouse model. Oncoimmunology.

[121] Sugiyama D, Nishikawa H, Maeda Y, Nishioka M, Tanemura A, Katayama I, Ezoe S, Kanakura Y, Sato E, Fukumori Y (2013). Anti-CCR4 mAb selectively depletes effector-type FoxP3 + CD4+ regulatory T cells, evoking antitumor immune responses in humans. Proc Natl Acad Sci U S A.

